# Potent and PPARα-independent anti-proliferative action of the hypolipidemic drug fenofibrate in VEGF-dependent angiosarcomas *in vitro*

**DOI:** 10.1038/s41598-019-42838-y

**Published:** 2019-04-19

**Authors:** Yasser Majeed, Rohit Upadhyay, Sara Alhousseiny, Tarek Taha, Adham Musthak, Yanal Shaheen, Mohtashim Jameel, Chris R. Triggle, Hong Ding

**Affiliations:** 1 Department of Pharmacology, Weill Cornell Medicine-Qatar, Education City, Doha 24144 Qatar; 2 Department of Medical Education, Weill Cornell Medicine-Qatar, Education City, Doha 24144 Qatar

**Keywords:** Cancer therapy, Sarcoma, Sarcoma

## Abstract

Angiosarcomas are highly aggressive tumors of endothelial origin, which carry a poor prognosis. Fenofibrate is a hypolipidemic drug, which acts by activating the transcription factor PPARα. It has also been widely reported to have ‘anti-cancer’ activity. The current study investigated its effect in a murine VEGF-dependent angiosarcoma cell-line, MS1 VEGF. The study utilised assays to monitor cell proliferation and viability, apoptosis, cell cycle progression, mitochondrial membrane potential, changes in protein expression, and changes in miRNA expression using microarrays. Fenofibrate showed potent anti-proliferative action in MS1 VEGF angiosarcoma cells, without inducing apoptosis. It enriched cells in G2/M cell cycle phase and hyperpolarised mitochondria. Other PPARα activators failed to mimic fenofibrate action. Inhibitors of PPARα and NFκB failed to reverse the inhibitory effect of fenofibrate and their combination with fenofibrate was cytotoxic. Fenofibrate downregulated the expression of key VEGF-effector proteins, including Akt, ERK, Bcl-2 and survivin, and a chemical inhibitor screen discovered relevance of these proteins to cell proliferation. A miRNA microarray revealed that fenofibrate differentially regulated cellular miRNAs with known roles in cancer and angiogenesis. The data raise the possibility that fenofibrate could be useful in angiosarcoma therapy, especially considering its well-established clinical safety and tolerability profile.

## Introduction

Cancer is one of the biggest challenges facing modern medicine, with estimates from the WHO indicating that 1 in 6 deaths globally can be attributed to cancer^1^. Importantly, emerging evidence links components of the metabolic syndrome including type 2 diabetes and obesity to an increase in the risk of developing cancer^2^. Tumor growth relies on a constant supply of nutrients and angiogenesis facilitates tumor growth and metastases^3,4^. Therefore, targeting angiogenesis is a rational therapeutic approach for combating malignancies. For example, antibodies targeting VEGF, such as bevacizumab, suppress angiogenesis by antagonizing growth factor signaling in endothelial cells and have demonstrated therapeutic efficacy in many cancers^4^. Nevertheless, targeting angiogenesis is associated with adverse effects, notably in the cardiovascular system^5^.

Angiosarcomas are rare and highly aggressive soft tissue malignancies of endothelial origin, which carry a poor prognosis^6^. They can appear sporadically or in association with radiation exposure or chronic lymphedema. Histological analysis of angiosarcomas in mice and humans revealed a central role for early/late-stage endothelial progenitor cells and possibly hematopoietic stem cells, although some species-specific differences were also detected^7,8^. Genetic analysis of angiosarcomas uncovered mutations in genes such as Myc, FLT4, KDR, PLCγ and PTPRB^9,10^, and emerging evidence also implicates miRNAs in angiosarcoma pathogenesis^11^. Wide local excision and adjunct radiotherapy are the mainstay treatment and although benefit has been reported using chemotherapeutics^12^, their severe side-effect profile makes their clinical impact questionable. The efficacy of anti-angiogenic therapies in angiosarcomas is also under investigation^13^ but incomplete understanding of pathological mechanisms has hindered progress in drug development.

Angiosarcoma studies have relied heavily on the use of *in vitro* systems including MS1 VEGF and MS1 SVR angiosarcoma cells, which show VEGF- and oncogenic H-Ras-dependent tumorigenicity, respectively^14,15^. These cells induce tumors *in vivo* that recapitulate the gross histology of angiosarcomas and have proved valuable for angiosarcoma studies and angiogenesis research in general. For example, Hasenstein *et al*. identified a role for tunica internal endothelial kinase 2 (Tie2) and vascular endothelial growth factor (VEGF) in promoting survival of MS1 VEGF and MS1 SVR cells^16^. Our lab identified inhibitory effects of metformin, albeit at millimolar concentrations, on endoplasmic reticulum stress and autophagy in MS1 VEGF angiosarcoma cells thus providing support to both the clinical and pre-clinical studies that infer an anti-cancer action for this anti-hyperglycemic drug^17^. The *in vivo* tumorigenic nature of MS1 VEGF cells therefore confers an advantage over the use of primary endothelial cells (e.g. HUVEC) to investigate angiogenesis mechanisms in cancer.

Fenofibrate is a cholesterol-lowering drug prescribed to patients at risk of cardiovascular disease and for the treatment of atherosclerosis and, furthermore, has an excellent efficacy and tolerability profile^18,19^. Fenofibrate is converted to its active metabolite fenofibric acid, which activates the transcription factor peroxisome proliferator-activated receptor alpha (PPARα). This stimulates lipoprotein lipase, lowers apoprotein CIII, and improves blood triglycerides and HDL-cholesterol levels^19^. In addition to its hypolipidemic action, it has also become apparent that fenofibrate exerts robust ‘anti-cancer’ activity and elicits inhibitory effects in several types of cancers, including lymphoma, glioblastoma, prostate and breast cancer^20–25^. Fenofibrate also protects against diabetic retinopathy^26^ and promotes angiogenesis in rodent models of ischemia^27^. Fenofibrate enhances AMPK and eNOS phosphorylation to reduce endothelial cell proliferation^28,29^ and its cytotoxicity in glioblastoma is associated with mitochondrial depolarization^23^. Fenofibrate therefore is now being repurposed to be part of an anti-angiogenic multidrug combination regimen for cancer therapy^30^. However, it is not known whether fenofibrate is effective in angiosarcomas and mechanisms underlying its anti-cancer actions require further exploration.

The current study was designed to determine whether fenofibrate when used within a concentration range comparable to that used clinically, possesses anti-proliferative actions in MS1 VEGF angiosarcoma cells. The results demonstrate that fenofibrate, without reducing cell viability or inducing apoptosis has potent anti-proliferative effects. The inhibitory effects were not replicated by other PPARα agonists and not reversed by antagonists of PPARα or NFκB. These effects were associated with downregulation of key oncoproteins and changes in expression of cancer-related cellular miRNAs. Collectively the data provide insight into a robust *in vitro* action of fenofibrate that could be used to advantage in angiosarcomas and other types of cancer.

## Results

### Potent suppression of MS1 VEGF angiosarcoma cell proliferation by fenofibrate

**Figure 1:**
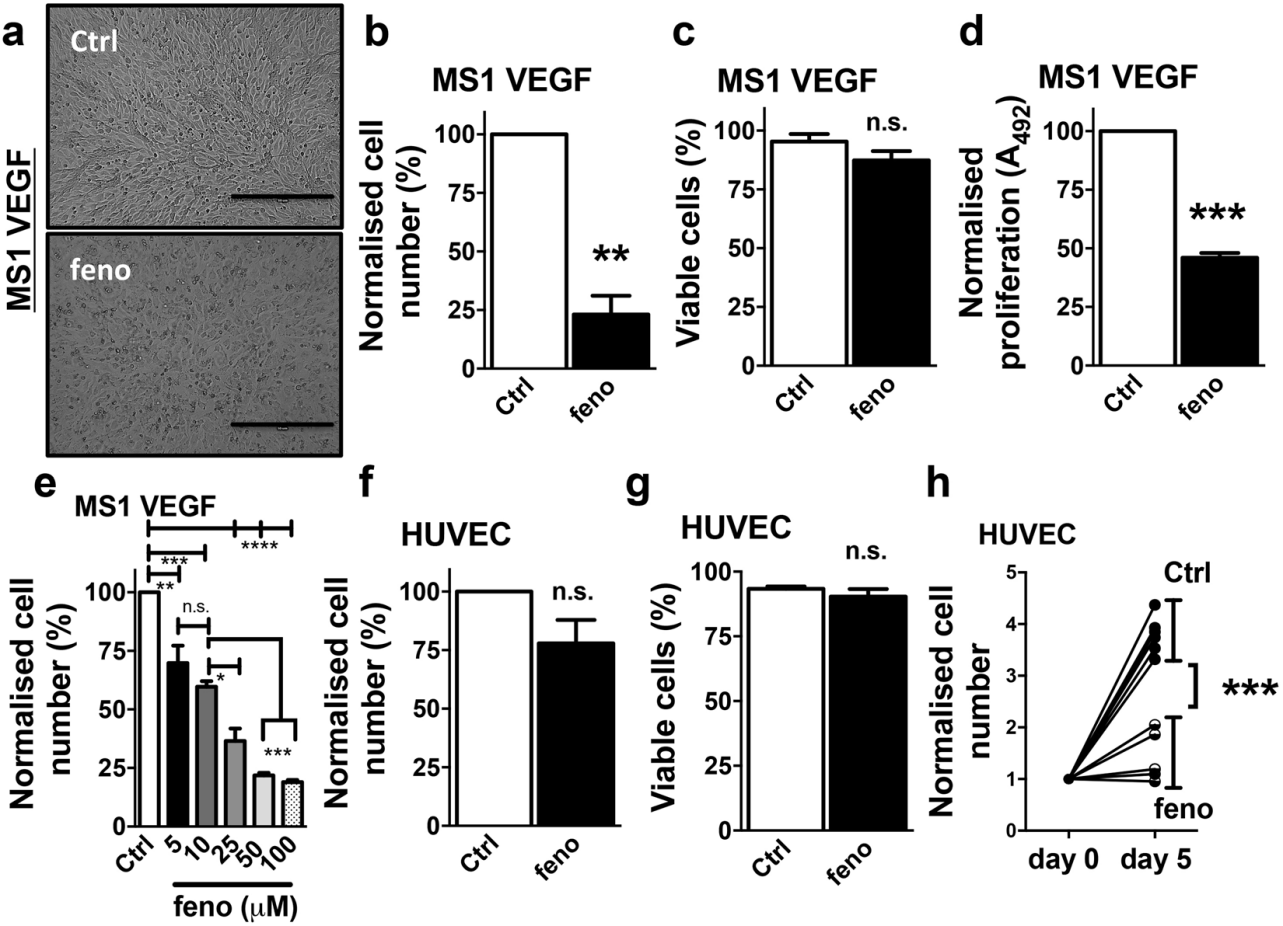
Fenofibrate inhibits MS1 VEGF angiosarcoma cell proliferation. Data were generated in MS1 VEGF angiosarcoma cells (**a**–**e**) or human umbilical vein endothelial cells (HUVEC, **f**–**h**). (**a**) Images of MS1 VEGF angiosarcoma cells under control conditions (Ctrl, DMSO-treated) or after treatment with 50 μM fenofibrate (feno) for 48 hours. Scale bar, 50 μm. (**b**–**d**) Effect of 48-hour treatment with 50 μM fenofibrate on cell number (b, n = 6), cell viability (**c**, n = 6) or cell proliferation determined by MTS assay (d, n = 4). (**e**) Concentration-dependent effect of fenofibrate (n = 3 for each concentration; one-way ANOVA followed by Bonferroni’s multiple comparisons test). (**f**,**g**) Effect of a 48-hour treatment with 50 μM fenofibrate on HUVEC cell number (**f**, n = 5) or viability (g, n = 5). (**h**) HUVEC proliferation rate data under control conditions (Ctrl, DMSO-treated) or after treatment with 50 μM fenofibrate for 5 days (n = 6). Each data point represents an independent experiment. Student’s *t*-test was used to analyse data shown in b-d and f-h. *P < 0.05; **P < 0.01; ***P < 0.001; ****P < 0.0001; n.s, not significant.

To test the effect of fenofibrate in MS1 VEGF angiosarcoma cells, cells were treated with 50 μM fenofibrate (or 0.1% DMSO) for 48 hours. These experiments revealed a robust decrease in cell number after fenofibrate treatment (~20 ± 5.3% of control) (Fig. 1a,b), without reducing cell viability (Control, 96.8 ± 1.9% *vs* fenofibrate, 91.40 ± 3.3%) (Fig. 1c). MTS proliferation assays also revealed a robust fenofibrate-induced reduction in MS1 VEGF angiosarcoma cell proliferation (~46.0 ± 2% of control) (Fig. 1d). To assess potency, concentration-response experiments were performed and these revealed relatively potent effects of fenofibrate, with cell proliferation reduced by concentrations ≥ 5 μM (Fig. 1e). Parallel comparative experiments were performed in human umbilical vein endothelial cells (HUVEC). Treatment with 50 μM fenofibrate for 48 hours did not affect HUVEC number or viability (Fig. 1f,g). However, considering the relatively slow proliferation rate of HUVEC, it was hypothesized that a possible inhibitory effect of fenofibrate may be unmasked by allowing HUVEC to proliferate for a longer duration. Indeed, the data suggested a 3.79 ± 0.14-fold increase in HUVEC cell number when cultured for 5 days. Treatment with 50 μM fenofibrate significantly suppressed this increase (fold increase ~1.39 ± 0.18), without reducing cell viability (Fig. 1h). Collectively, the experiments revealed that fenofibrate exerted potent anti-proliferative action in MS1 VEGF angiosarcoma cells, whereas HUVEC, exposed to 10-fold higher concentrations of fenofibrate were less affected.

### Fenofibrate did not induce apoptosis, but enriched cells in the G2/M cell cycle phase and hyperpolarised mitochondria in MS1 VEGF angiosarcoma cells

**Figure 2:**
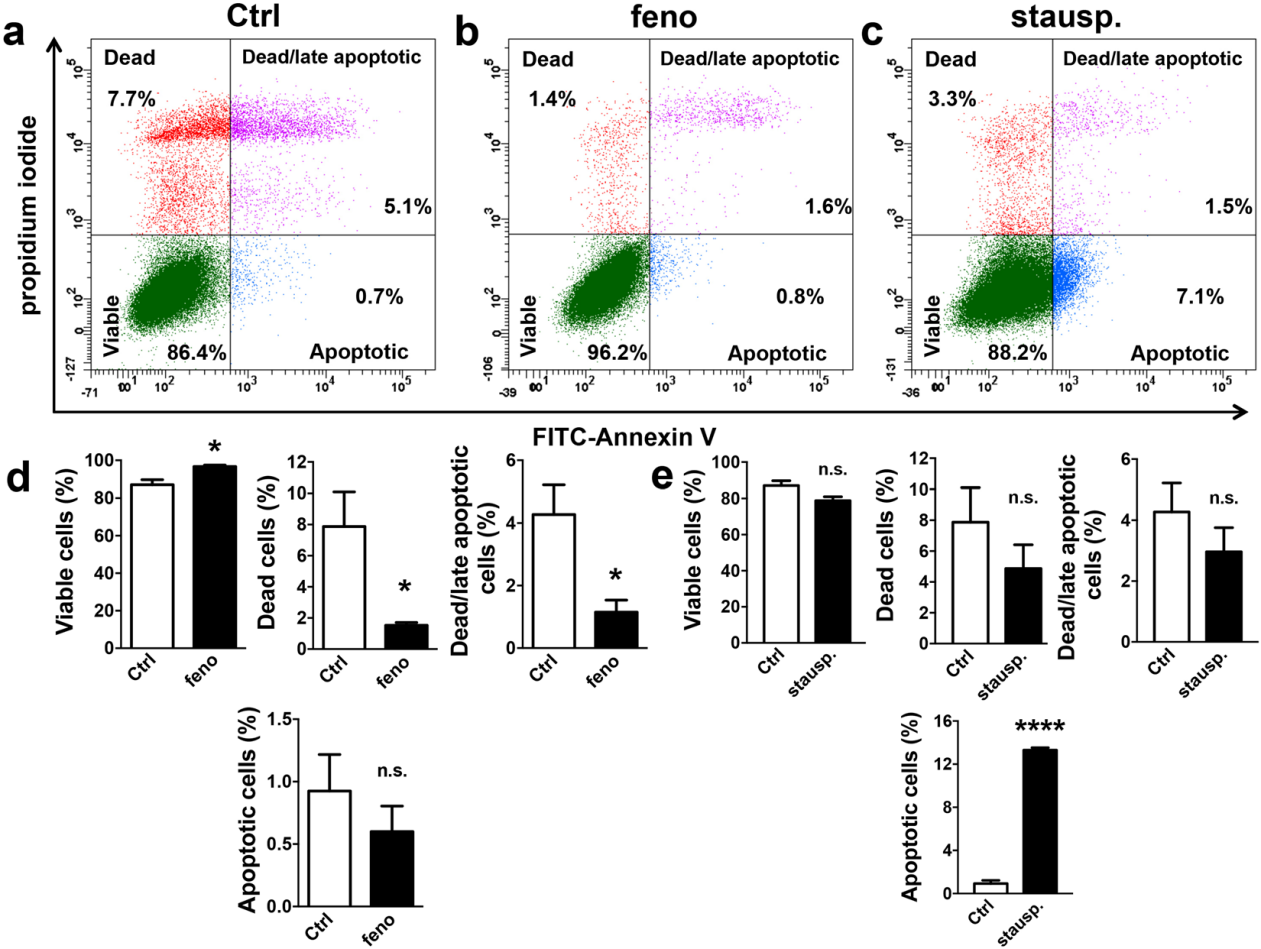
Fenofibrate did not induce apoptosis in MS1 VEGF angiosarcoma cells. Data were generated in MS1 VEGF angiosarcoma cells by flow cytometry. (**a**–**c**) Example experiment showing the proportion (%) of dead, early apoptotic, or dead/late apoptotic cells after treatment with 50 μM fenofibrate (feno) or 1 μM staurosporine (stausp). Staining for FITC-conjugated Annexin V was used as a marker for early apoptosis whereas propidium iodide (PI) staining was used as a marker for cell death. (**d**,**e**) Mean data for experiments exemplified in a-c (n = 4, fenofibrate data set; n = 3, staurosporine data set). Data were analysed using a Student’s *t*-test. *P < 0.05; ****P < 0.0001; n.s, not significant.

To investigate if the anti-proliferative action of fenofibrate in MS1 VEGF angiosarcoma cells was associated with early apoptosis, treated cells were stained with either FITC-conjugated Annexin V (early apoptosis) or propidium iodide (cell death) or both. Flow cytometry analysis revealed no significant increase in Annexin V staining after 48-hour treatment with 50 μM fenofibrate (Control, 0.92 ± 0.3% *vs* fenofibrate, 0.6 ± 0.2%). In contrast, staurosporine – included as a positive control - induced a robust apoptotic response, evidenced by increased Annexin V staining (13.3 ± 0.2%). There was also a small but significant improvement in cell viability (Control, 86.9 ± 2.6% *vs* fenofibrate, 96.7 ± 0.7%) and a significant decrease in the percentage of dead cells after fenofibrate treatment (Control, 7.8 ± 2.2% *vs* fenofibrate, 1.5 ± 0.2%) (Fig 2a–g).

**Figure 3:**
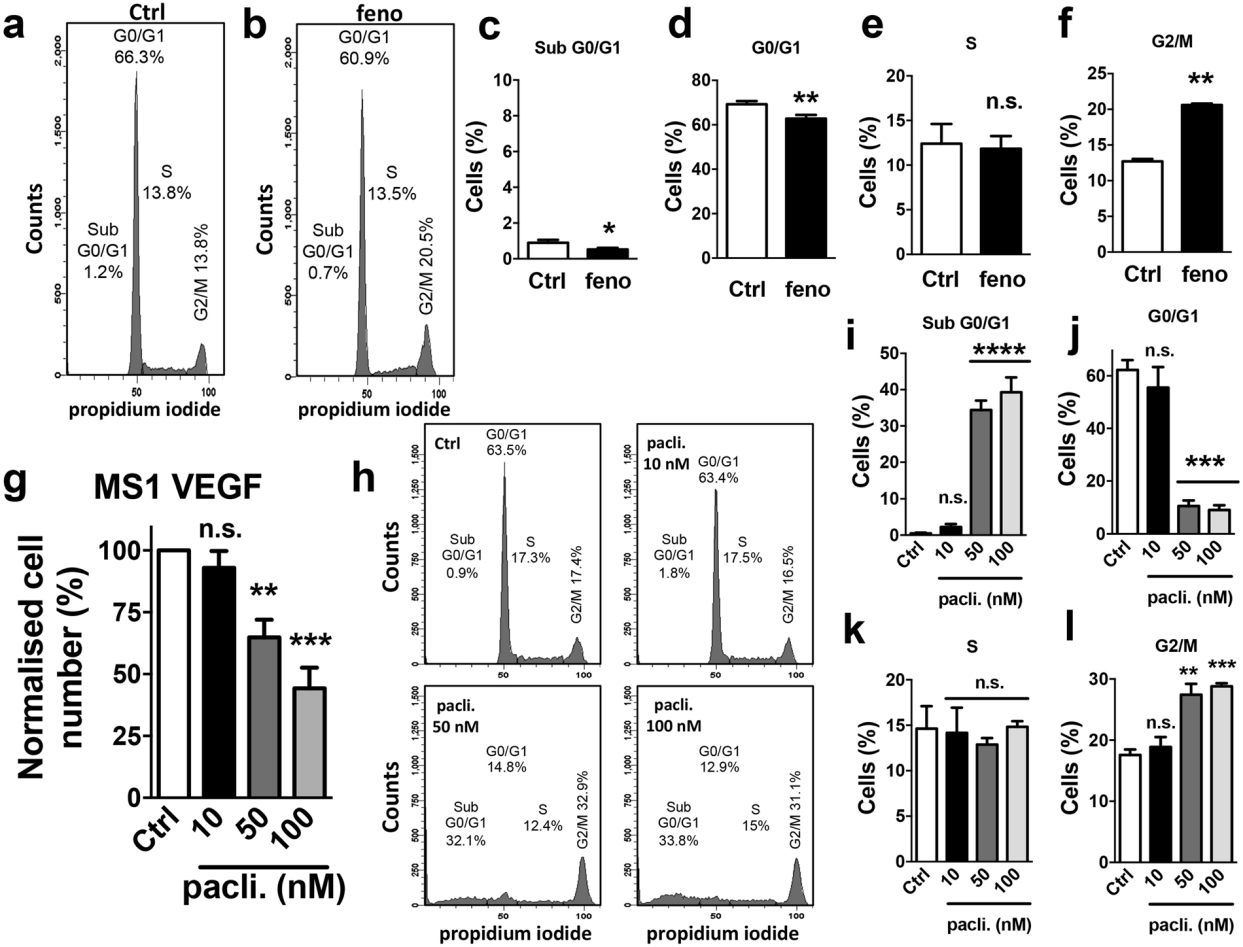
Fenofibrate- and paclitaxel-induced cell cycle changes in MS1 VEGF angiosarcoma cells. Cell cycle data were generated in MS1 VEGF angiosarcoma cells by flow cytometry. (**a**,**b**) Example experiment showing the effect of a 48-hour treatment with 50 μM fenofibrate (feno) on different cell cycle phases. Comparisons were made with DMSO-treated (Ctrl) cells. (**c**–**f**) Mean data for experiments exemplified in a,b (n = 4). Statistical analysis was performed using a Student’s *t*-test. (**g**) Mean data for the effect of different concentrations of paclitaxel on cell number (n = 4). (**h**–**l**) Example experiment showing the effect of different concentrations of paclitaxel on cell cycle (**h**) and mean data for the exemplified experiment (**i**–**l**) (n = 3). Statistical analysis was performed using one-way ANOVA followed by Dunnett’s multiple comparisons test. *P < 0.05; **P < 0.01; ***P < 0.001; ****P < 0.0001; n.s, not significant.

To investigate if fenofibrate triggered cell cycle arrest in MS1 VEGF angiosarcoma cells, treated cells were permeabilized and labeled with propidium iodide to distinguish G0/G1, S and G2/M phases. The data revealed that fenofibrate induced a significant decrease in the proportion of cells in the G0/G1 phase (Control, 69.2 ± 1.3% *vs* fenofibrate, 62.8 ± 1.6%) and an increase in the G2/M population (Control, 12.7 ± 0.4% *vs* fenofibrate, 20.6 ± 0.2%). The proportion of cells in the S phase was unaffected, however (Control, 12.4 ± 2.2% *vs* fenofibrate, 11.83 ± 1.4%) (Fig. 3a–f). The clinically used drug paclitaxel is known to induce G2/M arrest by inhibiting microtubule function. Experiments were therefore performed to compare the effect of paclitaxel with fenofibrate. Paclitaxel (50–100 nM but not 10 nM) significantly reduced MS1 VEGF angiosarcoma cell number to ~64.8 ± 7.2% (50 nM) and ~44.23 ± 8.4% (100 nM) of control, respectively (Fig. 3g). Cell cycle analysis revealed that paclitaxel (50 or 100 nM), like fenofibrate, significantly increased the proportion of cells in the G2/M phase (Control, 17.57 ± 0.9% *vs* 100 nM paclitaxel, 28.80 ± 0.5%). However, unlike fenofibrate, paclitaxel also caused a robust increase in the sub-G0/G1 population, indicating cytotoxicity (Control, 0.5 ± 0.2% *vs* 100 nM paclitaxel, 39.30 ± 4.1%) and a ‘collapse’ of the G0/G1 population (Control, 62.2 ± 3.7% *vs* 100 nM paclitaxel, 9.1 ± 1.8%) (Fig. 3h–l).

**Figure 4:**
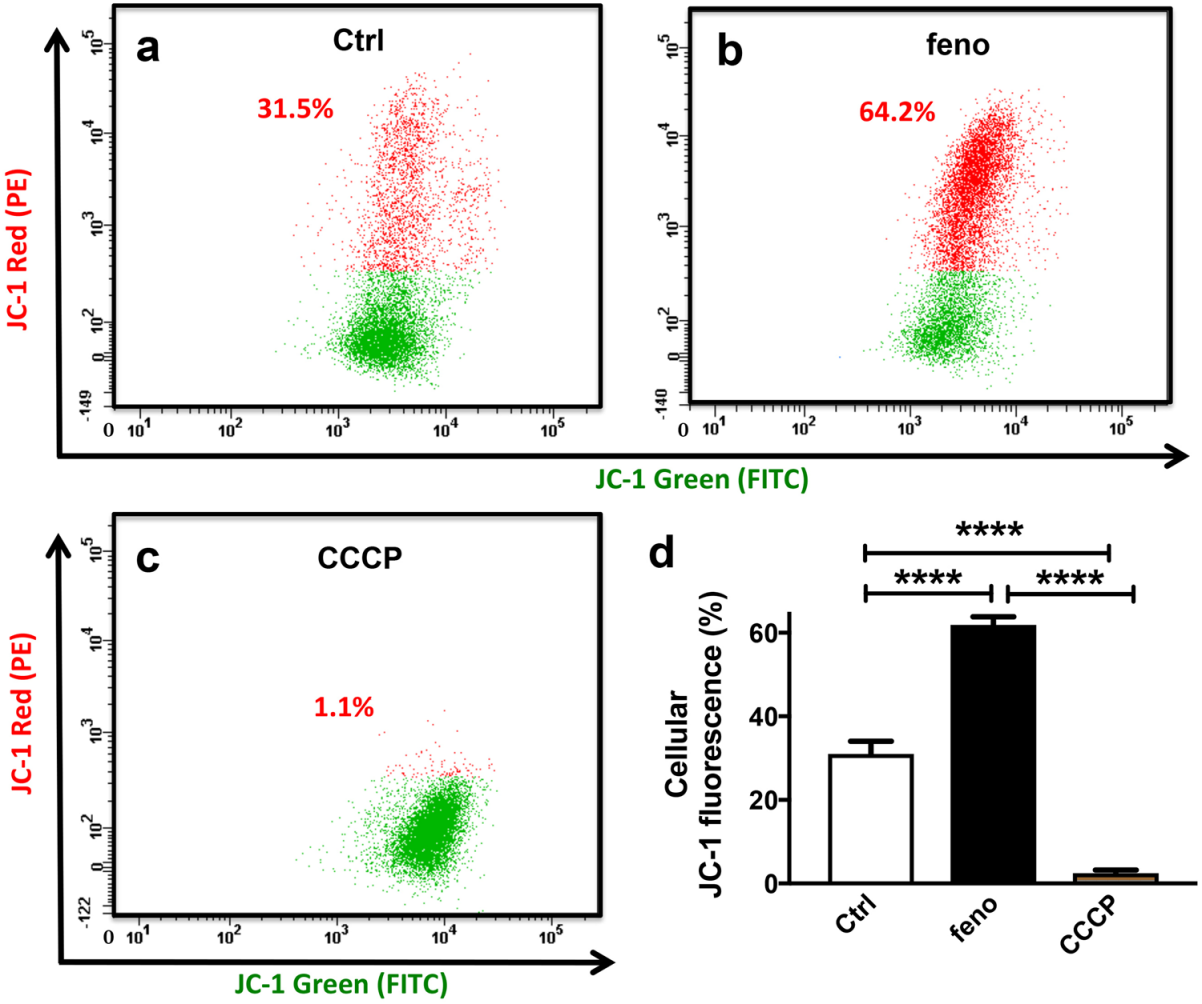
Hyperpolarized mitochondria in fenofibrate-treated MS1 VEGF angiosarcoma cells. Data were generated by flow cytometry in MS1 VEGF angiosarcoma cells using the mitochondrial membrane potential indicator dye JC-1. (**a**–**d**) Example experiment showing the effect of a 48-hour treatment with DMSO (Ctrl) or 50 μM fenofibrate on JC-1 red fluorescence (**a**,**b**). The mitochondrial depolarizing agent CCCP was used as a positive control (**c**). (**d**) Mean data for experiments exemplified in a-c (n = 4). ****P < 0.0001 (one-way ANOVA followed by Tukey’s multiple comparisons test).

To investigate if fenofibrate modulated mitochondrial membrane potential in MS1 VEGF angiosarcoma cells, experiments were performed using the mitochondrial membrane potential-sensitive dye JC-1, which forms red aggregates upon accumulation in hyperpolarized mitochondria. The data revealed that JC-1 Red fluorescence was enhanced by ~2-fold after 48-hour treatment with 50 μM fenofibrate. In contrast, this signal was virtually abolished in cells treated with the mitochondrial depolarizing agent CCCP, which was included as a positive control (JC-1 Red fluorescence: Control, 31.0 ± 3.0% *vs* fenofibrate, 61.8 ± 1.9% *vs* CCCP, 2.5 ± 0.8%) (Fig. 4a–d).

### PPAR alpha- and NFκB-independent action of fenofibrate in MS1 VEGF angiosarcoma cells

**Figure 5:**
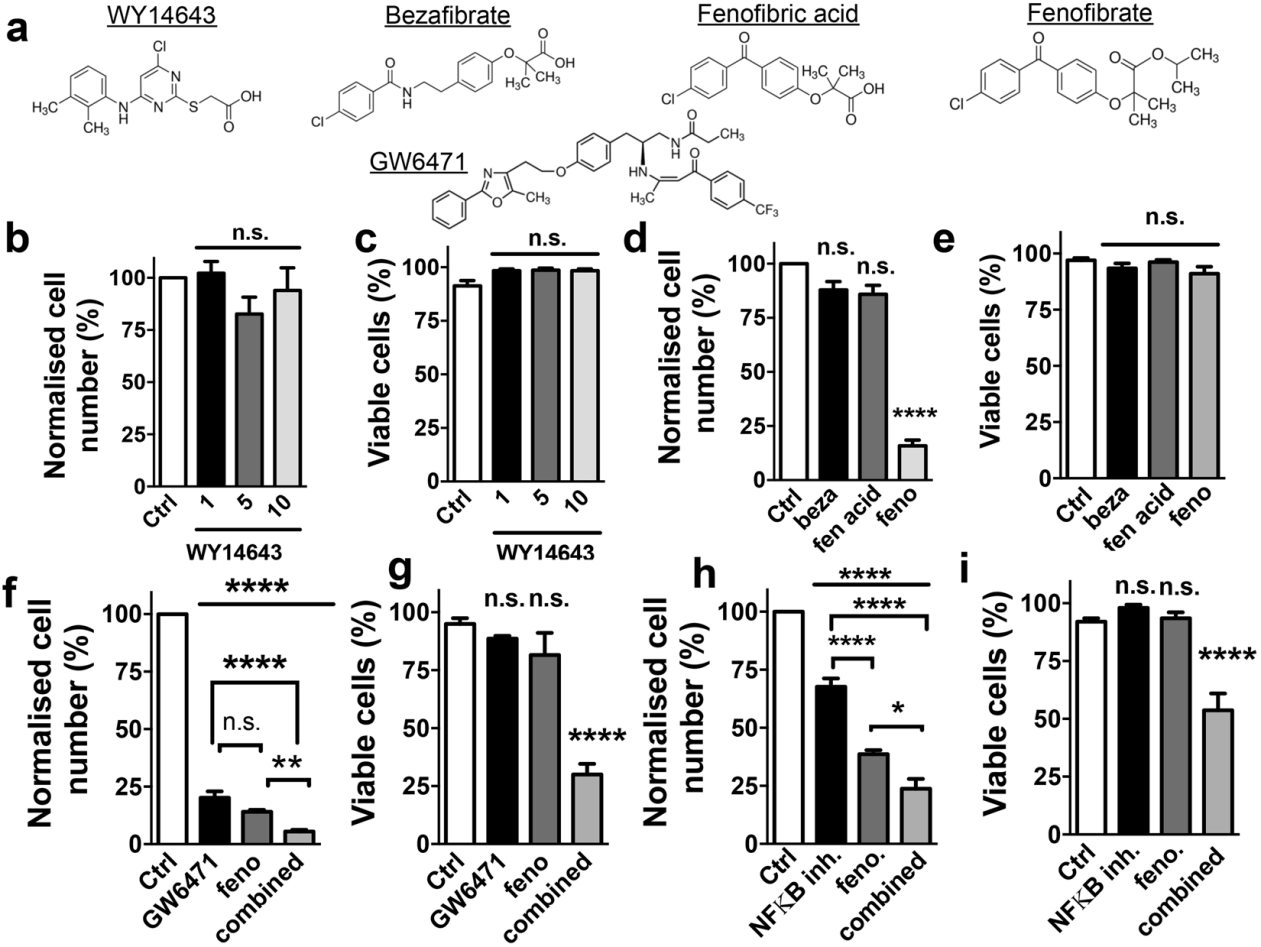
PPARα- and NFκB-independence of fenofibrate action in MS1 VEGF angiosarcoma cells. (**a**) Chemical structures of the PPARα agonists (WY14643, bezafibrate, fenofibric acid and fenofibrate) and the PPARα antagonist GW6471. (**b**,**c**) Mean data for the effect of a 48-hour treatment with the indicated concentrations of WY14643 on cell proliferation and viability (n = 4). (**d**,**e**) Mean data for the effect of a 48- hour treatment with 50 μM each of bezafibrate (beza), fenofibric acid (fen. acid) or fenofibrate (feno) on cell proliferation and viability (n = 4). (**f**,**g**) Mean data for the effect of a 48-hour treatment with 10 μM GW6471, 50 μM fenofibrate or their combination on cell proliferation and viability (n = 4). (**h**,**i**) Mean data for the effect of a 24-hour treatment with 10 μM PDTC (NFκB inhibitor), 50 μM fenofibrate or their combination on cell proliferation and viability (n = 4). Statistical analysis was performed using one-way ANOVA followed by Tukey’s multiple comparisons test. *P < 0.05; **P < 0.01; ***P < 0.001; ****P < 0.0001; n.s, not significant.

To gain mechanistic insight into the anti-proliferative action of fenofibrate in MS1 VEGF angiosarcoma cells and to test the relevance of PPARα and NFκB, experiments were performed using: **(a)** WY14643 - potent and specific PPARα agonist **(b)** bezafibrate and fenofibric acid **(c)** GW6471 - PPARα antagonist and **(d)** PDTC - NFκB inhibitor (Fig. 5a). These experiments revealed that neither WY14643 (1–10 μM), bezafibrate (50 μM) nor fenofibric acid (50 μM) inhibited cell proliferation. Fenofibrate - included as a positive control - strongly reduced cell proliferation (~15.8 ± 2.6% of control) (Fig. 5b–e). To test if GW6471 reversed the action of fenofibrate, cells were incubated with this antagonist (10 μM) for 1 hour followed by treatment with 50 μM fenofibrate in the presence of the antagonist. Treatments with GW6471 or fenofibrate alone were also performed. As expected, fenofibrate strongly reduced cell number (~14.1 ± 0.8% of control). Cell number was also reduced by GW6471 (~20.22 ± 2.7% of control), without affecting cell viability. Furthermore, the combination of GW6471 with fenofibrate reduced cell number further (to ~5.5 ± 0.8% of control) and reduced cell viability (Control, ~95.0 ± 2.4% *vs* combination, ~30.0 ± 4.5%). Loss of cell viability was not observed with GW6471 alone (~88.6 ± 1.2% viability) or fenofibrate alone (~81.6 ± 9.5% viability) (Fig. 5f,g). Similar experiments were performed using PDTC, but over 24 hours because of cytotoxicity observed with longer PDTC treatments. Under these conditions, both 50 μM fenofibrate (~38.6 ± 1.7% of control) and 10 μM PDTC significantly reduced cell number (~67.7 ± 3.5% of control). In addition, the combination of PDTC and fenofibrate further reduced cell number (~23.7 ± 4.2% of control) and robustly decreased cell viability (Control, 92 ± 1.5% *vs* combination, 53.8 ± 7.2%). Cytotoxicity was not observed with PDTC alone (~98 ± 1.3% viability) or fenofibrate alone (~93.5 ± 2.5% viability) (Fig. 5h,i).

### Fenofibrate-induced down-regulation of ‘oncoproteins’ and relevance to MS1 VEGF angiosarcoma cell proliferation

**Figure 6:**
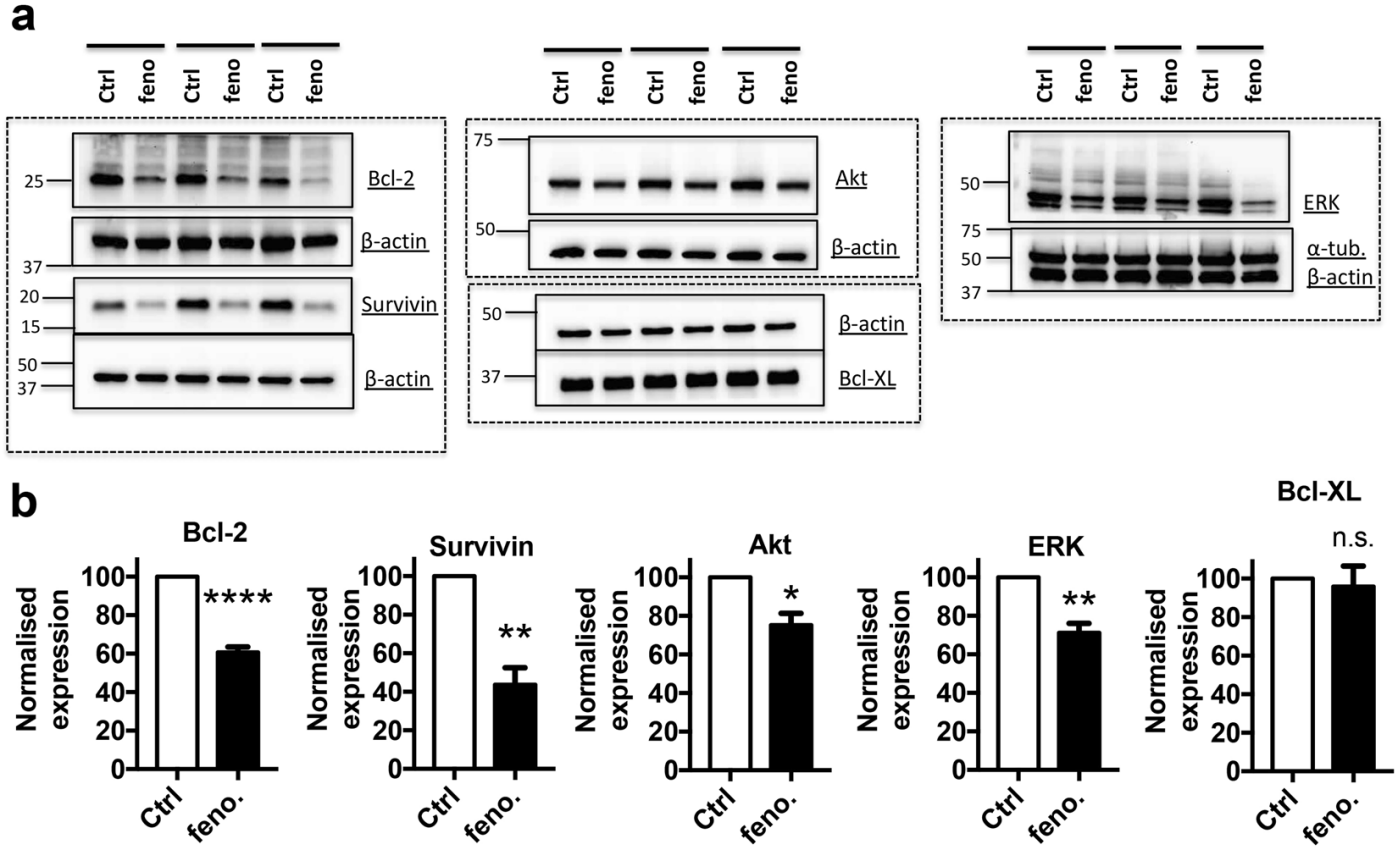
Fenofibrate down-regulates ‘oncoproteins’ in MS1 VEGF angiosarcoma cells. Data were generated by western blotting in MS1 VEGF angiosarcoma cells. (**a**) Example experiments (horizontal bars) showing the effect of a 48-hour treatment with 50 μM fenofibrate (feno) on the expression of Bcl-2, survivin, Akt, ERK, Bcl-XL, α- tubulin and β-actin. (**b**) Mean data from 5–6 independent experiments for data exemplified in (**a**). β-actin was used as a normalization control. Dashed boxes indicate individual gels and boxed regions within correspond to different sections of the same gel. *P < 0.05; **P < 0.01; ****P < 0.0001; n.s., not significant (Student’s *t*-test).

MS1 VEGF angiosarcoma cells exhibit VEGF-dependent tumorigenicity and proteins such as Akt, ERK, Bcl-2 and survivin are known ‘VEGF-effectors’. To gain further mechanistic insight into the anti-proliferative effect of fenofibrate, the drug’s impact on these key proteins was investigated by western blotting. Expression of Akt, ERK, Bcl-2 and survivin was readily detected in MS1 VEGF angiosarcoma cells (Fig. 6a). Importantly, fenofibrate treatment significantly reduced their expression to (% of control): Akt, 75.1 ± 6.1%; Bcl-2, 60.7 ± 2.7%; survivin, 43.7 ± 8.8%; and ERK, 71.2 ± 4.9%. Expression of other proteins such as Bcl-XL, α-tubulin and β-actin was largely unaltered by fenofibrate (Fig. 6a,b), thus indicating that the reductions in the expression levels of Akt, Bcl-2, survivin, and ERK were not non-specific effects of fenofibrate.

**Figure 7:**
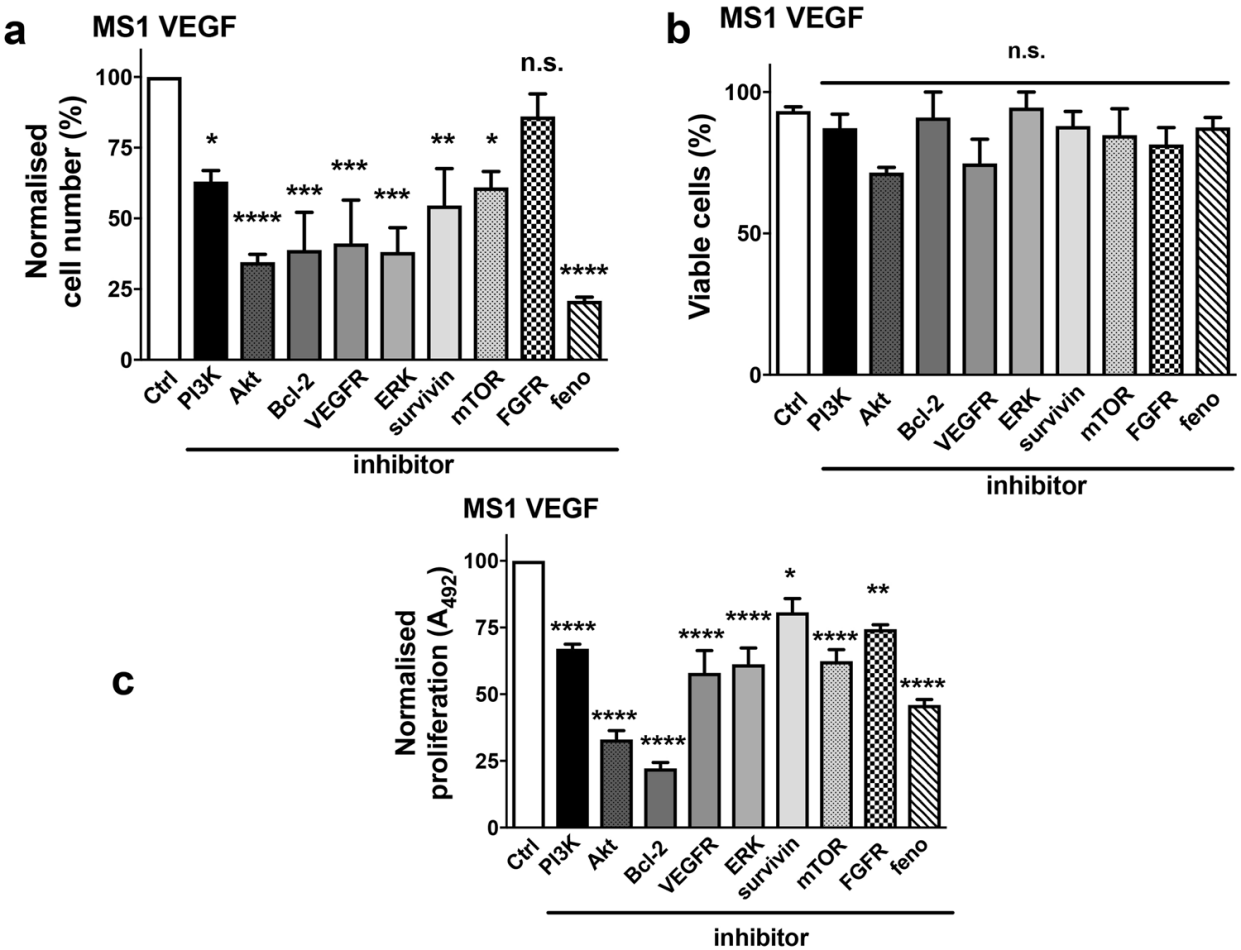
Chemical inhibitor screen identifies oncoproteins relevant to MS1 VEGF angiosarcoma cell proliferation and survival. (**a**–**c**) Mean data showing the effect of various chemical inhibitors of the indicated proteins on cell number (**a**), viability (**b**), and proliferation determined by MTS assay (n = 4). The chemical inhibitors (and concentrations) used were: PI3K - LY294002 (10 μM), Akt - Akt1/2 kinase inhibitor (10 μM), Bcl-2 - TW37 (1 μM), VEGFR - SU1498 (10 μM), ERK - PD98059 (10 μM), survivin - YM155 (1 μM), mTOR - temsirolimus (1 μM), and FGFR – SU5402 (10 μM). Fenofibrate (50 μM) was used as a positive control in the screen. Statistical analysis was performed using one-way ANOVA followed by Dunnett’s multiple comparisons test. *P < 0.05; **P < 0.01; ***P < 0.001; ****P < 0.0001; n.s, not significant.

To evaluate if ‘oncoprotein’ down-regulation by fenofibrate could be functionally important, a chemical screen using relatively specific inhibitors was performed in MS1 VEGF angiosarcoma cells. These experiments revealed anti-proliferative effects with several inhibitors, including 10 μM LY294002 (PI3K inhibitor), 10 μM Akt1/2 kinase inhibitor, 1 μM TW-37 (Bcl-2 inhibitor), 10 μM SU1498 (VEGF Receptor antagonist), 10 μM PD98059 (ERK inhibitor), 1 μM YM155 (survivin inhibitor), and 1 μM temsirolimus (mTOR inhibitor). The cell counts were (% of control): LY294002, 63.0 ± 3.9; Akt1/2 kinase inhibitor, 34.5 ± 2.7; TW-37, 38.8 ± 13.3; SU1498, 41.1 ± 15.3; PD98059, 38.13 ± 8.5; YM155, 54.5 ± 13.1; and temsirolimus, 60.98 ± 5.6 (Fig. 7a). In contrast, the FGF Receptor inhibitor SU5402 (10 μM) lacked effect. Importantly, 50 μM fenofibrate was tested in parallel and it strongly reduced cell number (to ~20.9 ± 1.3% of control). None of the inhibitors significantly reduced cell viability (Fig. 7b). MTS proliferation assays were performed using the same set of inhibitors. The data revealed that all inhibitors significantly reduced cell proliferation to (% of control): LY294002, 67.0 ± 1.6; Akt1/2 kinase inhibitor, 33.0 ± 3.3; TW-37, 22.2 ± 2.2; SU1498, 57.9 ± 8.3; PD98059, 61.2 ± 6.1; YM155, 80.7 ± 5.1; and temsirolimus, 62.3 ± 4.3 (Fig. 7c).

### Expression profiling of cancer-relevant miRNAs in MS1 VEGF angiosarcoma cells and fenofibrate-induced changes in expression

**Figure 8:**
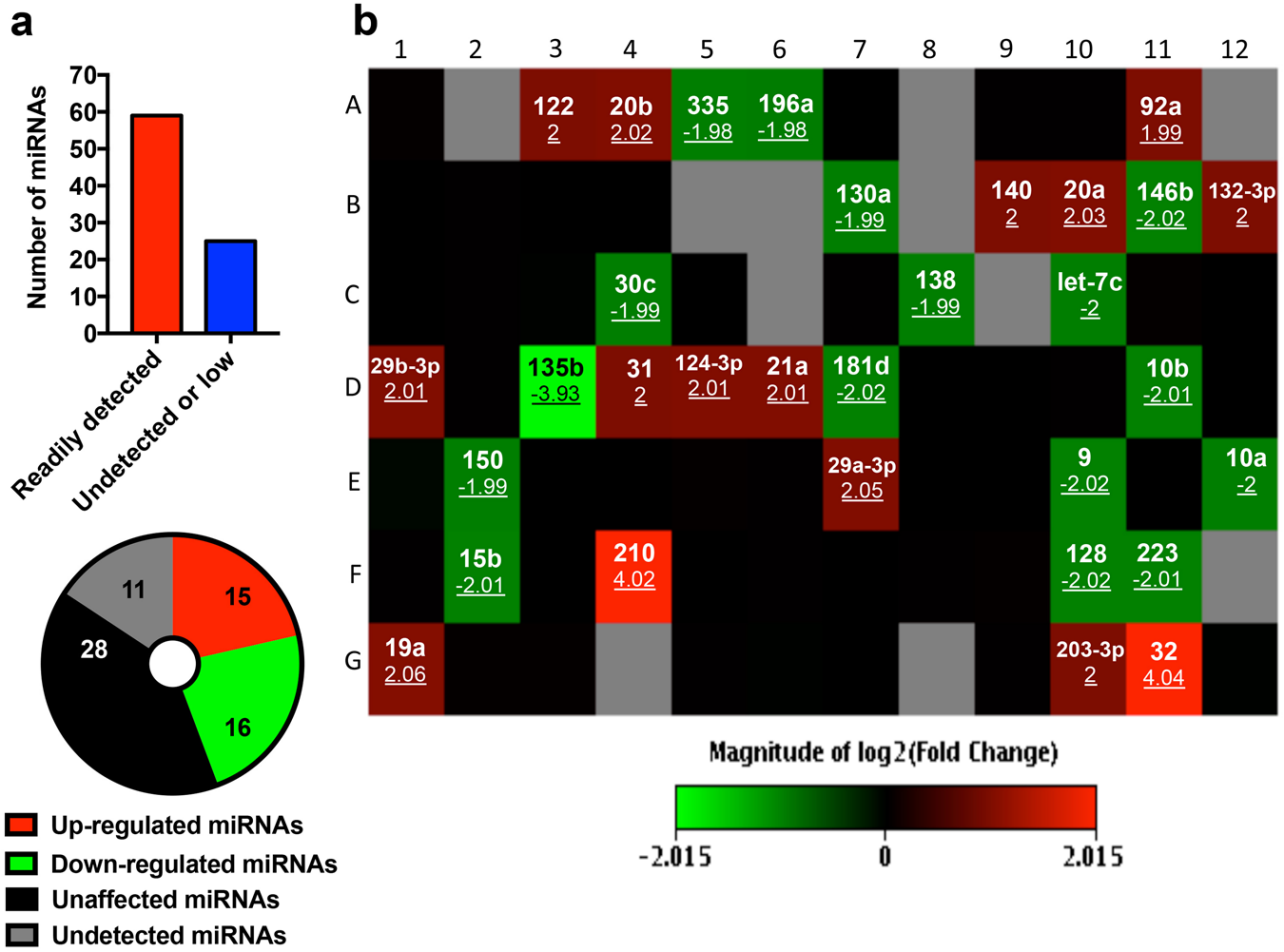
Fenofibrate-induced alterations in cancer-related microRNAs in MS1 VEGF angiosarcoma cells. (**a**) Pie chart showing the miRNA expression data, categorized as either readily detected (C_T _ <30), low expression (C_T_ > 30) or undetected (C_T_ > 35), and miRNAs up-regulated, down-regulated or unchanged after treatment with fenofibrate. C_T_, Cycle Threshold. (**b**) Microarray data profiling changes in 84 cancer- relevant microRNAs after treatment with 50 μM fenofibrate for 48 hours. The color- coded boxes indicate microRNAs whose expression was unchanged (black), up- regulated or down-regulated (green) by fenofibrate. The gray boxes indicate miRNAs that were not detected in the array. The miRNA ID and the fold-change in expression (underlined) are also indicated. The array was performed in samples pooled from 3 independent experiments.

MicroRNAs (miRNAs) are highly conserved non-coding RNA molecules that regulate gene expression, thereby controlling cell proliferation and survival in cancer. Widespread reduction in expression of oncoproteins by fenofibrate prompted us to investigate if changes in cellular miRNAs could be associated with this effect. To this end, a miRNA microarray was performed and 84 miRNAs with established roles in cancer were evaluated. Expression of 59 miRNAs was readily detected, which included miRNAs such as miR20b-5p, miR92a-3p, miR130a-3p, miR-let-7c-5p and miR-let-7e-5p (Fig. 8, Supplementary Table 1). Of these 59, expression of 28 miRNAs was not altered by fenofibrate (black squares), 15 miRNAs were up-regulated (red squares), and 16 miRNAs were down-regulated (green squares) (Supplementary Table 1 and Fig. 8). The miRNAs that were up-regulated (~2–4 fold) included miR210, miR32-5p, miR122-5p, miR20b-5p, miR92a-3p, miR140-5p and miR20a-5p. miRNAs that were down-regulated (~2-fold) by fenofibrate treatment included miR196a-5p, miR130a-3p, miR10b-5p, miR150-5p, and miR9-5p (Fig. 8b).

## Discussion

Although the effects of fenofibrate in primary endothelial cells have been well-studied, there is little known about its actions in angiosarcomas. Data from the current study illustrate that fenofibrate exerted potent anti-proliferative actions in MS1 VEGF angiosarcoma cells, which were independent of PPARα and NFκB. Fenofibrate neither reduced cell viability nor induced apoptosis, arrested cells in G2/M phase, hyperpolarized mitochondria, and downregulated key VEGF-dependent ‘oncoproteins’. An inhibitor screen revealed functional relevance of these oncoproteins to angiosarcoma cell proliferation. In addition, a miRNA microarray screen uncovered robust fenofibrate-induced changes in cellular miRNAs, many of which have known roles in angiogenesis and cell proliferation.

Previous studies have reported that fenofibrate suppresses angiogenesis^31,32^, reduces endothelial tube formation^33^, and suppresses proliferation by inducing a G0/G1 block^29^. It also suppresses angiogenesis *in vivo* via a PPARα-dependent mechanism but accelerates wound healing in diabetic mice^31,32,34,35^. Concentration-response studies of fenofibrate in MDA-MB-231 cells revealed an ~IC_50_ of 16 µM for apoptosis-induction^22^. In contrast, our data revealed an apparent IC_50_ of 8 µM for fenofibrate’s anti-proliferative action in MS1 VEGF angiosarcoma cells (Fig. 1e), which fits well with the plasma concentrations reported in clinical use^36,37^. In contrast to this study, fenofibrate was reported to depolarize mitochondria and trigger apoptosis in glioblastoma^23,38^. These observations suggest that modulation of mitochondrial membrane potential by fenofibrate is cell-type dependent. Importantly, normal human astrocytes were less sensitive to fenofibrate when compared to glioblastoma cells, although fenofibrate suppressed mitochondrial respiration in both cell-types^23^. This observation suggests that alternative mechanisms potentially mediate the inhibitory effects of fenofibrate in cancer cells. The data presented in this study raise the possibility that fenofibrate-mediated changes in cellular miRNAs and oncoprotein downregulation could play an important role. Comparative histological analysis of primary tumors from mice and humans suggested that human angiosarcomas arise from bone-marrow derived hematopoietic stem cells or early EPC, whereas in mice early EPCs appear to play a major role^7,8^. The clinical relevance of the effect of fenofibrate in angiosarcomas could therefore be supported by testing its effects in cells isolated from such tumors. Furthermore, studies comparing the efficacy and potency of fenofibrate in mouse *versus* human angiosarcoma cells will also be informative and could potentially reveal species-specific differences in fenofibrate action. Importantly, these studies also suggest involvement of VEGF signaling, which could be relevant because we revealed a suppressive effect of fenofibrate on key VEGF-related oncoproteins.

Structure-activity studies using other PPARα agonists revealed that bezafibrate, WY14643 and fenofibric acid were all ineffective. These data not only revealed the PPARα-independence of fenofibrate action but also how minor changes in structure profoundly affected activity. For example, fenofibrate - but not bezafibrate or WY14643 – protected endothelial cells from apoptosis^39^ and WY14643 failed to replicate the inhibitory effect of fenofibrate on mitochondrial respiration^23^. Fenofibrate and fenofibric acid differ only slightly in chemical composition (Fig. 5), but only fenofibrate was effective. This cannot be explained by reduced membrane permeability of fenofibric acid because it is readily-permeable and was shown, for example, to protect epithelial cells against high glucose-induced damage^40^. There are few reports directly comparing the actions of fenofibrate and fenofibric acid, although differential effects on AMPK^28^ and 11β-hydroxysteroid dehydrogenase have been reported^41^. The latter study also observed that esterification/amidation of the carboxy group was essential for activity, which may be relevant to our study. AMPK activation however is unlikely to explain the anti-proliferative action of fenofibrate because bezafibrate also activates AMPK^42^ but was ineffective in MS1 VEGF angiosarcoma cells. When used individually, GW6471 (PPARα antagonist) and PDTC (NFκB antagonist) reduced cell proliferation without affecting cell viability but their combination with fenofibrate was cytotoxic. These data not only support the argument that the effects of fenofibrate in MS1 VEGF cells are independent of signaling via the PPARα and NFκB pathways, but also that, inhibiting both pathways results in cytotoxicity. Reversal of fenofibrate effects by GW6471 or PDTC in cancer cells has been reported^22,33,35,38^ but, to our knowledge, their cytotoxicity when combined with fenofibrate is a novel observation. NFκB promotes a Warburg effect in pediatric sarcomas^43^, so it is conceivable that the combination of glycolysis-inhibition (by PDTC) combined with downregulation of ‘survival’ proteins (by fenofibrate) triggers MS1 VEGF angiosarcoma cell death. Inhibition of glycolysis with GW6471 has also been reported^44^. Cytotoxic effects of a combination of fenofibrate with glycolysis inhibitors have been reported and our lab discovered a similar mechanism for metformin cytotoxicity in MS1 VEGF angiosarcoma cells^17,45^. Further studies will be required to clarify the underlying mechanisms and pharmacological studies supporting PPARα- and NFκB-independence may be complemented by RNA interference (RNAi)-mediated ‘knock-down’ experiments.

Biochemical investigations revealed that treatment with fenofibrate significantly reduced the expression of oncoproteins like Akt, survivin, Bcl-2 and ERK. A chemical screen of inhibitors targeting these oncoproteins uncovered effects of several chemicals, including Akt1/2 inhibitor, YM155 (survivin), TW37 (Bcl-2), and PD98059. Importantly, these experiments also revealed that fenofibrate was the most effective agent among all tested compounds. Although this may be explained by concentration-dependence in some cases, it is also possible that the high efficacy is due to fenofibrate’s ability to down-regulate these key proteins simultaneously. There is little known about the relevance of these molecules to murine angiosarcomas, although they have been investigated in human/canine angiosarcomas. For example, survivin was overexpressed in human angiosarcomas and YM155 inhibited proliferation in human angiosarcoma cells^46^. Constitutive activation of the PI3K-Akt-mTOR signaling pathway was also reported in human angiosarcomas^47^. Furthermore, canine angiosarcomas showed constitutive ERK activation and MEK inhibition reduced *in vitro* cell viability^48^. Fenofibrate-induced growth arrest in G2/M phase coupled to downregulation of anti-apoptotic proteins Bcl-2 and survivin may also sensitize MS1 VEGF angiosarcoma cells to cytotoxic agents^49^. The data therefore confirm the established oncogenic roles for proteins like Akt, survivin, Bcl-2 and ERK in angiosarcomas and expand the pharmacological utility of specific inhibitors to murine VEGF-dependent angiosarcomas.

The relevance of miRNAs in angiosarcomas and soft tissue sarcomas is starting to be elucidated. For example, miR-497-5p, -378-3p and 483-5 were downregulated in angiosarcoma and targeting of a potassium channel (KCa3.1) by miR-497-5p led to inhibition of cell proliferation and invasion^50^. Sarver *et al*. profiled the expression of miRNAs in over 20 different sarcomas and reported significant upregulation of chromosome 19 miRNAs in angiosarcomas relative to other sarcomas^51^. Concepcion *et al*. reported Myc-dependent expression of the miR-17-92 cluster, which may be relevant to angiosarcomas that develop secondary to radiation exposure^52,53^. There is therefore considerable interest in identifying miRNAs with functional and therapeutic relevance to angiosarcomas. In our study, we profiled fenofibrate-induced changes in cellular miRNAs and identified several miRNAs that were differentially expressed. Although little is known about the relevance of these miRNAs to angiosarcomas, there is evidence in the literature suggesting that many of the upregulated miRNAs exert anti-angiogenic and anti-proliferative roles in cancer. These miRNAs include miR122, 140-5p and -20b, which are known to target VEGF^54–56^. miRNA-210 was robustly induced by fenofibrate in our study and has been shown to also reduce proliferation and induce G2/M arrest in colorectal cancer cells^57^ and its overexpression was associated with improved prognosis^58^. Furthermore, fenofibrate-induced miRNAs known to target Akt, survivin, Bcl-2 and ERK include miR29b, -29a-3p and -122 (Akt)^59^, miR31 (Bcl-2)^60^, miR203 (survivin)^61^ and miR20b^62^. Among the miRNAs downregulated by fenofibrate in MS1 VEGF angiosarcoma cells, many are known drivers of cancer cell proliferation, including miR335, -146, -130a, and -135b^63–65^. The data therefore raise the intriguing possibility that the anti-proliferative effect of fenofibrate in MS1 VEGF angiosarcoma cells may at least partly be driven by differential changes in cellular miRNAs with pro- or anti-proliferative activity. A direct way to test this hypothesis would be to artificially manipulate the expression of individual or a combination of miRNAs using miRNA mimics or inhibitors to evaluate their role in cell proliferation and understand if this manipulation alters the efficacy and/or potency of fenofibrate.

In conclusion, we report potent inhibitory effects of the cholesterol-lowering drug fenofibrate in MS1 VEGF angiosarcoma cells, which were independent of PPARα and NFκB. Combined treatment with fenofibrate and a PPARα- or NFκB antagonist led to cytotoxicity. Fenofibrate downregulated the expression of Akt, survivin, ERK and Bcl-2 and a chemical screen uncovered a role for these ‘oncoproteins’ in cell proliferation and viability. Finally, this study discovered that fenofibrate induces robust changes in cellular miRNAs, many of which potentially regulate angiogenesis and have established roles in cancer. The data therefore establish fenofibrate as a potent inhibitor of VEGF-dependent angiosarcoma cell proliferation and highlight important pharmacological differences with its observed effects in other cancer cells in terms of potency, effects on apoptosis and mitochondrial function, PPARα- and NFκB-dependence, and interactions with PPARα- and NFκB pathways. The drug may have potential utility in angiosarcoma therapy.

## Materials and Methods

### Cell culture

MS1 VEGF angiosarcoma cells were purchased from American Type Culture Collection (ATCC^R^ CRL-2460^TM^). Cells were grown in Dulbecco’s Modified Eagle’s Medium (DMEM, Cat# 11885, Invitrogen), supplemented with 5% fetal bovine serum (Cat# F2442, Sigma Aldrich) and 1% antibiotics (penicillin/streptomycin, Cat# 15140, LifeTechnologies). The total glucose concentration in the medium was adjusted to 11 mM (equivalent to non-fasting basal blood glucose in non-diabetic mice) using a 10% glucose solution (Cat# G8644, Sigma Aldrich). MS1 VEGF angiosarcoma cells were passaged every 2–3 days and used for experiments up to passage 15. 80–90% confluent cells were washed once with PBS and then incubated with trypsin (Cat# T3924, Sigma Aldrich) for 5 minutes to detach cells. Trypsin was neutralized using cell culture medium, the cells were mixed well by pipetting, transferred to a 15 ml polypropylene tube, and centrifuged at 300 g for 5 minutes. The supernatant was removed and the cells were re-suspended in cell culture medium. Human Umbilical Vein Endothelial Cells (HUVEC) were purchased either from ATCC (PCS 100-010^TM^) or Lonza (Cat# C2519A). HUVECs were grown in Medium 199 (M199, Cat# M4530, Sigma Aldrich) supplemented with 15% fetal bovine serum (Cat# F6178, Sigma Aldrich), 30 μg/ml Endothelial Cell Growth Supplement (ECGS, Cat# 356006, BD Biosciences) and 100 μg/ml heparin (Cat# H4784, Sigma Aldrich). Cells were used for experiments up to passage 6. For HUVEC culture, Detachin (Cat# T100100, Genlantis) was used instead of trypsin and cells were not centrifuged. Both MS1 VEGF angiosarcoma cells and HUVECs were grown in a 37 °C humidified incubator supplied with 5% CO_2_.

### Drug treatments

Fenofibrate (Cat# F6020, Sigma Aldrich) was dissolved in 100% anhydrous dimethyl sulfoxide (DMSO) and was prepared fresh on the day of the experiment. The drug was always prepared at 1000x concentration (e.g. 50 mM) and then diluted 1:1000 in cell culture medium to achieve the desired final concentration (e.g. 50 μM). The solution was mixed well by pipetting to aid solubility before addition to the cells. In most experiments, the total duration of treatment with fenofibrate was 48 hours and the treatment medium was replenished after 24 hours. The method of preparation and treatment protocol for the other PPARα agonists were the same as those described for fenofibrate. In experiments involving GW6471 (PPARα antagonist) and PDTC (NFκB antagonist), cells were pre-treated with each antagonist for 1 hour prior to treatment with fenofibrate and the antagonist was maintained throughout the course of the experiment. For apoptosis assays, cells were treated with staurosporine for 3 hours prior to analysis. In JC-1 mitochondrial membrane potential measurement experiments, cells were treated with the mitochondrial depolarizing agent carbonyl cyanide m-chlorophenyl hydrazine (CCCP) for 30 minutes and the agent was also included during the JC-1 dye loading step.

### Cell counts and viability analysis

For cell counts, 10 μl of the cell suspension was mixed with 10 μl trypan blue (Cat# 1450021, Bio-Rad). 10 μl of this mixture was then added to a Dual Chamber Cell Counting Slide (Cat# 145-0011) and counts were made using a TC20^TM^ Automated Cell Counter (Bio-Rad). Trypan blue exclusion was used to assess cell viability and determine the live/dead cell count.

### Proliferation assay

Cell proliferation assays were performed using the CellTiter 96^R^ Aqueous One Solution Proliferation Assay (MTS), according to the manufacturer’s instructions. Briefly, cells were grown in 96-well plates and treated with drugs in 100 μl cell culture medium. At the end of the treatment period, 20 μl of the CellTiter 96^R^ Aqueous One Solution Reagent was added to each well, mixed by pipetting and incubated for 3 hours in a humidified, 5% CO_2_ incubator. The absorbance was then recorded at 490 nm using a 96-well plate reader. Wells without cells but incubated with the reagent (with culture medium) were used to determine background non-specific absorbance, which was corrected for during analysis.

### Apoptosis assay

Apoptosis assays were performed using the FITC Annexin V Apoptosis Detection Kit I (Cat# 556547, BD Pharmingen) following the manufacturer’s protocol. Briefly, treated cells were centrifuged (300 g, 5 minutes), washed once in cold PBS and then re-suspended in 1x Binding Buffer. The resuspended cells (100 μl) were incubated with either propidium iodide or FITC Annexin V (single-stained) or both (double-stained) for 15 minutes in the dark at room temperature. The final volume of the suspension was adjusted to 500 μl using 1x Binding Buffer and analysis was performed within 1 hour using a BD LSR Fortessa Cell analyzer (BD Biosciences). Unstained and single-stained samples were used as compensation controls during the experiment. Staff providing technical support during this experiment (and other flow cytometry experiments) was blinded to the experimental groups to limit bias during data collection and analysis.

### Cell cycle analysis

Treated cells were re-suspended in PBS following trypsinization and centrifugation steps as described above in the cell culture section. Cells were then fixed by adding this suspension drop-wise to ice-cold 100% ethanol and stored overnight at −20 °C. Fixed cells were stained using a solution that contained (in PBS): 50 μg/ml propidium iodide (Cat# P1304MP, LifeTechnologies), 100 μg/ml RNase A (Cat# 12091-021, LifeTechnologies) and 0.1% Triton-X 100 (Cat# A16046, Alfa Aesar). Cells were stained for 1 hour in a 37 °C water bath, washed once with PBS and re-suspended in 500 μl PBS for analysis. Unstained cells were used as control during the experiment.

### JC-1 mitochondrial membrane potential assay

Mitochondrial membrane potential measurements were made using the MitoProbe^TM^ JC-1 Assay Kit for Flow Cytometry (Cat# M34152, Life Technologies). Treated cells were detached using trypsin and the cell suspension was centrifuged at 300 g for 5 minutes. The pellet was re-suspended in warm cell culture medium containing 2 μM JC-1 dye and incubated in a humidified, 5% CO_2_ incubator at 37 °C for 30 minutes. 2 ml cell culture medium was then added, followed by centrifugation at 300 g for 5 minutes. The pellet was re-suspended in 400 μl PBS and analyzed immediately on a BD LSR Fortessa Cell analyzer (BD Biosciences). Cells not stained with JC-1 and those stained with JC-1 but treated with the mitochondrial depolarizing agent carbonyl cyanide m-chlorophenyl hydrazine (CCCP) were used as controls during the experiment.

### Western blotting

Western blotting experiments were performed using standard protocols. Briefly, cell lysates were prepared in RIPA buffer supplemented with a protease/phosphatase inhibitor cocktail (1:100, Cat# 1861284, ThermoScientific) and EDTA (1:100). 30–40 μg proteins were separated on an SDS-PAGE gel followed by transfer onto a nitrocellulose membrane. Membranes were ‘blocked’ with 5% bovine serum albumin dissolved in TRIS-buffered saline (TBS) containing 0.1% Tween 20 (wash buffer). Incubation with the primary antibody was overnight at 4 °C on a rocker. Following 3 washes, membranes were incubated for 1 hour at room temperature with the appropriate HRP-conjugated secondary antibodies. Following 3 washes, proteins were detected using the Amersham ECL Prime Western Blotting Detection Reagent (Cat# RPN2232, GE Healthcare Life Sciences) and visualized on a Geliance P600 Gel Documentation System (PerkinElmer, Inc. MA, USA). The band densities of individual proteins were quantified using the Bio-Rad Quantity One software. All antibodies were purchased from Cell Signaling Technology (CST). The primary antibodies and dilutions used were: Akt (1:1000, Cat# 4691), Bcl-2 (1:1000, Cat# 2870), ERK1/2 (1:1000, Cat# 9102), survivin (1:1000, Cat# 2808), Bcl-XL (1:1000, Cat# 2764), mTOR (1:1000, Cat# 2983), tubulin (1:1000, Cat# 2144) and β-actin (1:10,000, Cat# 3770). The secondary antibodies and dilutions used were: Anti-rabbit HRP-conjugated secondary antibody (1:3000, Cat# 7074) and anti-mouse HRP-conjugated secondary antibody (1:10,000, Cat# 7076).

### Chemical inhibitor screen

The chemical inhibitor screen was performed in either 12-well plates (cell counts/viability) or 96-well plates (MTS proliferation assays). Cells were treated with the inhibitors for a total duration of 48 hours (except TW-37, 24-hour treatment), and the treatment media was replenished after 24 hours. The inhibitors and concentrations used were: LY294002 (PI3K inhibitor, 10 μM), Akt1/2 kinase inhibitor (10 μM), TW-37 (Bcl-2 inhibitor, 1 μM), SU1498 (VEGFR inhibitor, 10 μM), PD98059 (ERK inhibitor, 10 μM), YM155 (survivin inhibitor, 1 μM), temsirolimus (mTOR inhibitor, 1 μM), and SU5402 (FGFR inhibitor, 10 μM). Fenofibrate was used as a positive control in all experiments. The supplier information for each inhibitor can be found in the Materials section.

### MicroRNA microarray

Expression profiling of mature microRNAs (miRNA) and changes in response to fenofibrate were analyzed using the Mouse miScript miRNA Cancer PathwayFinder PCR array (Cat# MIMM-102Z, Qiagen). The array profiles 84 miRNAs relevant to cancer and includes positive, negative and normalization controls. Cells were treated with fenofibrate as described above. RNA was extracted from samples pooled from 3 independent experiments using the miRNeasy Mini Kit (Cat# 217004, Qiagen) and 250 ng RNA was reverse-transcribed using the miScript II RT kit (Cat# 218160, Qiagen). 200 μl RNase-free water was added to dilute the cDNA prior to use. The reaction mix for the miRNA PCR array was prepared according to the manufacturer’s protocol and contained: 2X QuantiTect SYBR Green PCR Master, 10X miScript Universal Primer, RNase-free water, and template cDNA. 25 μl of this reaction mix was added to each well of the 96-well array plate and the plate centrifuged for 1 minute at 1000 g prior to PCR. The cycling conditions for real-time PCR were: Initial activation Step – 15 minutes, 95 °C; 3-step cycling (40 cycles) – Denaturation (15 seconds, 94 °C), Annealing (30 seconds, 55 °C) and Extension (30 seconds, 70 °C). Data were collected and after defining the fluorescence baseline and threshold, analysis was performed using the ΔΔC_T_ method of relative quantification using the online data analysis software available at http://pcrdataanalysis.sabiosciences.com/mirna. SNORD61 and SNORD96A were used as normalization controls and their expression was unchanged after treatment with fenofibrate. Changes in expression of miRNAs in response to fenofibrate treatment were plotted as a Heat Map depicted in Fig. 8.

### Data and statistical analysis

Data were analyzed using GraphPad Prism 7.0 and presented as mean ± SEM. Statistical analysis was performed using either a Student’s *t*-test (for 2 groups) or ordinary one-way ANOVA (followed by post-hoc analysis) when more than 2 groups were being compared. A ‘p’ value of less than 0.05 was considered statistically significant. Statistical analysis was only performed on data generated from independent experiments. Specific details of the statistical tests and experimental ‘n’ values are indicated in the legend of each figure.

### Materials

The following reagents were obtained from Sigma: fenofibrate (Cat# F6020), bezafibrate (Cat# B7273), fenofibric acid (Cat# 796565), GW6471 (Cat# G5045), PDTC (Cat# P8765), paclitaxel (Cat# T1912), LY294002 (Cat# L9908), SU5402 (Cat# SML0443), temsirolimus (Cat# PZ0020) and PD98059 (Cat# P215). The other reagents used in this study were: staurosporine (Cat# 9953, Cell Signaling Technology), WY14643 (Cat# 70730, Cayman Chemicals), TW-37 (Cat# 4038, Tocris Bioscience), YM155 (Cat# 11490, Cayman Chemicals), SU1498 (Cat# 572888, Calbiochem), propidium iodide (Cat# P1304MP, LifeTechnologies), RNase A (Cat# 12091-021, LifeTechnologies), and Triton-X 100 (Cat# A16046, Alfa Aesar).

## Supplementary information

Supplementary Data

## Data Availability

The datasets generated during the current study are available from the corresponding author on request.
