# Supplementary material for: Potent and PPARα-independent anti-proliferative action of the hypolipidemic drug fenofibrate in VEGF-dependent angiosarcomas in vitro
Source: Sci Rep. 2019 Apr 19;9:6316. doi: 10.1038/s41598-019-42838-y (PMC6474884; doi:10.1038/s41598-019-42838-y)
Supplement: Supplementary file 1 — Supplementary Data [file 41598_2019_42838_MOESM1_ESM.pdf]

SUPPLEMENTARY INFORMATION

Potent and PPARα-independent anti-proliferative action of the hypolipidemic drug fenofibrate in VEGF-dependent angiosarcomas *in vitro*

Yasser Majeed[1,*], Rohit Upadhyay[1], Sara Alhousseiny[1, 2], Tarek Taha[1, 2], Adham Musthak[1, 2], Yanal Shaheen[1, 2], Mohtashim Jameel[1], Chris R Triggle[1, 2], Hong Ding[1, 2 *]

[1]Department of Pharmacology and [2]Department of Medical Education, Weill Cornell Medicine-Qatar, Education City, Doha, Qatar, PO Box 24144.

*** Authors for correspondence:**

Dr Yasser Majeed, Postdoctoral Associate in Pharmacology, Department of Pharmacology, Weill Cornell Medicine-Qatar, Doha, Qatar, PO Box 24144. Telephone: (+974)-44928934. E-mail: yam2013@qatar-med.cornell.edu

(or)

Dr Hong Ding, Associate Professor of Research in Pharmacology, Department of Pharmacology, Weill Cornell Medicine-Qatar, Doha, Qatar, PO Box 24144. Telephone: (+974)-44928362. E-mail: hod2005@qatar-med.cornell.edu

Supplementary Table: Microarray to detect fenofibrate-induced changes in cancer-relevant miRNAs. The table includes the list of 84 miRNAs and their assignment within the 96-well microarray plate. Target expression (C_T) values in Control (0.1% DMSO)- and fenofibrate-treated cells are indicated, in addition to the calculated fold change. SNORD96A was used as the endogenous control for data normalization.

WELL	Mature ID	Ctrl	fenofibrate	Fold change
A01	mmu-let-7a-5p	24.93588257	24.91277122	1.02
A02	mmu-miR-133b-3p	35.93977356	35.93517303	1
A03	mmu-miR-122-5p	34.91236877	33.91579437	2
A04	mmu-miR-20b-5p	27.92950058	26.91789055	2.02
A05	mmu-miR-335-5p	26.92457199	27.91700554	-1.98
A06	mmu-miR-196a-5p	30.93502235	31.92500496	-1.98
A07	mmu-miR-125a-5p	22.92657089	22.9295845	1
A8	mmu-miR-142a-5p	Undetermined	Undetermined	1
A09	mmu-miR-96-5p	33.92160797	33.91548538	1.01
A10	mmu-miR-222-3p	24.93669128	24.92152977	1.01
A11	mmu-miR-92a-3p	26.90890312	25.92240715	1.99
A12	mmu-miR-184-3p	Undetermined	Undetermined	1
B01	mmu-miR-15a-5p	28.93863487	28.94211388	1
B02	mmu-miR-106a-5p	26.93645096	26.92916298	1.01
B03	mmu-miR-378a-3p	28.92695808	28.92671776	1
B04	mmu-let-7b-5p	25.93050003	25.93578529	-1
B05	mmu-miR-205-5p	36.97610092	36.90951538	1
B06	mmu-miR-137-3p	36.04257584	37.05566406	1
B07	mmu-miR-130a-3p	25.92522621	26.92311096	-1.99
B08	mmu-miR-199a-5p	37.06684875	35.92062759	1
B09	mmu-miR-140-5p	29.91273499	28.91936302	2
B10	mmu-miR-20a-5p	25.9346714	24.91477585	2.03
B11	mmu-miR-146b-5p	29.90658569	30.9265995	-2.02
B12	mmu-miR-132-3p	32.90467834	31.90969276	2
C01	mmu-miR-193b-3p	34.93021011	34.93456268	-1
C02	mmu-miR-183-5p	31.92201614	31.9174118	1.01
C03	mmu-miR-34c-5p	29.9124279	29.92506218	-1.01
C04	mmu-miR-30c-5p	21.93475914	22.93477249	-1.99
C05	mmu-miR-148a-3p	29.92855835	29.93034935	1
C06	mmu-miR-134-5p	37.08755493	35.93980408	1
C07	mmu-let-7g-5p	24.92316628	24.918396	1.01
C08	mmu-miR-138-5p	26.91534805	27.90878296	-1.99
C09	mmu-miR-141-3p	Undetermined	36.92817688	1
C10	mmu-let-7c-5p	23.90778542	24.91333389	-2
C11	mmu-let-7e-5p	24.9557209	24.92352295	1.03
C12	mmu-miR-218-5p	28.92085838	28.92638016	-1
D01	mmu-miR-29b-3p	27.91798401	26.9121151	2.01
D02	mmu-miR-146a-5p	30.9341011	30.92422676	1.01
D03	mmu-miR-135b-5p	30.93478203	32.91255951	-3.93

D04	mmu-miR-31-5p	23.91101074	22.91385651	2
D05	mmu-miR-124-3p	33.92910767	32.92904663	2.01
D06	mmu-miR-21a-5p	20.92340279	19.9220047	2.01
D07	mmu-miR-181d-5p	27.91039467	28.9313755	-2.02
D08	mmu-miR-301a-3p	25.92218208	25.9250412	1
D09	mmu-miR-195a-5p	22.92193222	22.92303848	1
D10	mmu-miR-100-5p	28.93616867	28.92419815	1.01
D11	mmu-miR-10b-5p	23.90637589	24.91918564	-2.01
D12	mmu-miR-155-5p	25.92173386	25.92570114	-1
E01	mmu-miR-1a-3p	34.94862747	35.94289398	-1.03
E02	mmu-miR-150-5p	26.93079376	27.92480659	-1.99
E03	mmu-let-7i-5p	24.92438316	24.92171097	1
E04	mmu-miR-27b-3p	23.91738701	23.92375183	-1
E05	mmu-miR-7a-5p	27.92990112	27.90505981	1.02
E06	mmu-miR-221-3p	24.93365288	24.92340469	1.01
E07	mmu-miR-29a-3p	22.94110298	21.91106033	2.05
E08	mmu-miR-191-5p	25.92211723	25.92600441	-1
E09	mmu-let-7d-5p	24.91808319	24.9145813	1.01
E10	mmu-miR-9-5p	24.93434906	25.95051193	-2.02
E11	mmu-let-7f-5p	23.91887665	23.92042351	1
E12	mmu-miR-10a-5p	24.92476273	25.92919731	-2
F01	mmu-miR-181b-5p	28.93732643	28.92929459	1.01
F02	mmu-miR-15b-5p	23.91397476	24.92730331	-2.01
F03	mmu-miR-16-5p	22.93417931	22.93452454	1
F04	mmu-miR-210-3p	31.94335365	29.94071007	4.02
F05	mmu-miR-17-5p	25.93621063	25.94267082	-1
F06	mmu-miR-98-5p	26.9298439	26.92365074	1.01
F07	mmu-miR-34a-5p	26.92129326	26.91951942	1
F08	mmu-miR-25-3p	24.93368912	24.92198181	1.01
F09	mmu-miR-144-3p	36.92528152	34.96735001	1.03
F10	mmu-miR-128-3p	28.91131973	29.92587471	-2.02
F11	mmu-miR-223-3p	33.91579437	34.92480087	-2.01
F12	mmu-miR-215-5p	36.9511261	37.01885605	1
G01	mmu-miR-19a-3p	25.95835876	24.9184227	2.06
G02	mmu-miR-193a-5p	30.96591568	30.93407631	1.02
G03	mmu-miR-18a-5p	27.94054222	27.91716766	1.02
G04	mmu-miR-214-3p	36.91039658	36.94837189	1
G05	mmu-miR-126a-3p	18.94328499	18.93671036	1.01
G06	mmu-miR-27a-3p	22.93947601	22.96047592	-1.01
G07	mmu-miR-103-3p	25.92319298	25.92969513	-1
G08	mmu-miR-149-5p	36.90130997	35.91169357	1

G09	mmu-miR-23b-3p	24.92694664	24.91783524	1.01
G10	mmu-miR-203-3p	34.91667557	33.920578	2
G11	mmu-miR-32-5p	34.92429352	32.91352463	4.04
G12	mmu-miR-181c-5p	28.899086	28.92258263	-1.01
H01	cel-miR-39-3p	36.92234421	Undetermined	1
H02	cel-miR-39-3p	Undetermined	Undetermined	1
**H03**	**SNORD61**	**25.94545174**	**25.96386147**	**-1.01**
H04	SNORD68	21.97171593	20.94314384	2.05
H05	SNORD72	24.94703865	23.91949463	2.04
H06	SNORD95	23.93820572	22.93654633	2.01
**H07**	**SNORD96A**	**21.93294525**	**21.92205238**	**1.01**
H08	RNU6-6P	21.9321785	23.91828346	-3.95
H09	miRTC	25.93450928	25.94080734	-1
H10	miRTC	25.92940521	25.93654251	-1
H11	PPC	20.90458488	20.90866852	-1
H12	PPC	20.89523315	20.90574265	-1

RUN 1

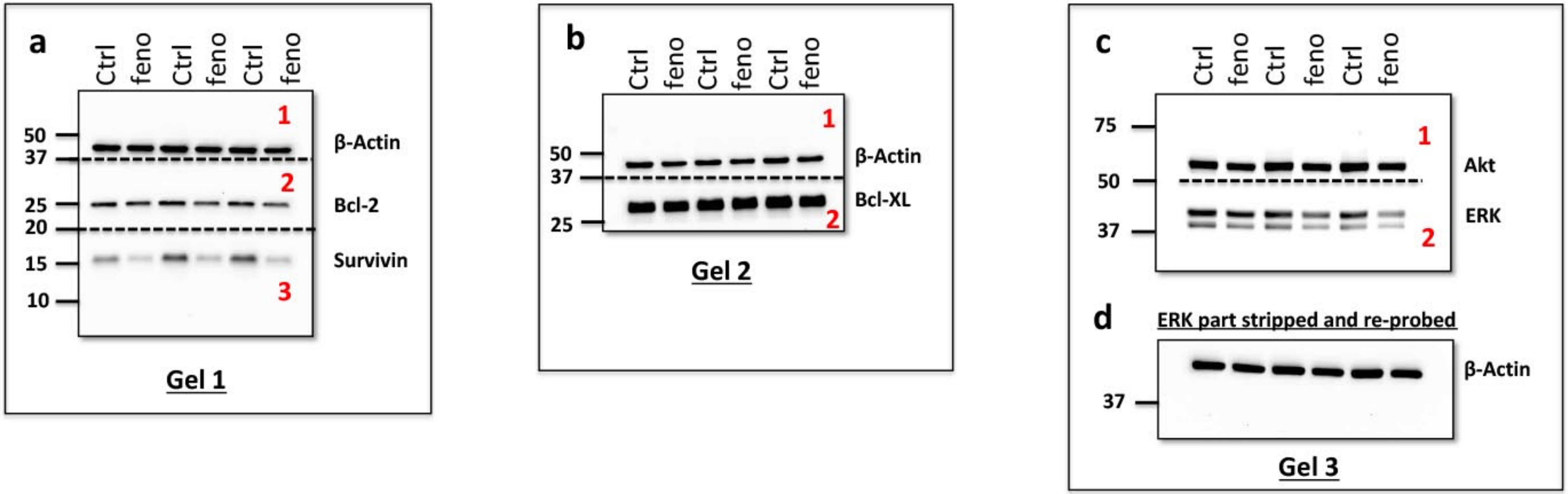

In Run 1 (Gels 1, 2 and 3), Control and fenofibrate-treated cell lysates from 3 independent experiments were prepared and analyzed by western blotting. Following transfer, membrane for Gel 1 was divided into 3 parts and probed for β-actin, Bcl-2 and survivin (as indicated) (a). Membrane for Gel 2 was divided into 2 parts and probed for β-actin and Bcl-XL (b). Membrane for Gel 3 was divided into 2 parts and probed for Akt and ERK (c). The 'ERK part' of the membrane was then stripped and re-probed for β-actin (d).

RUN 2

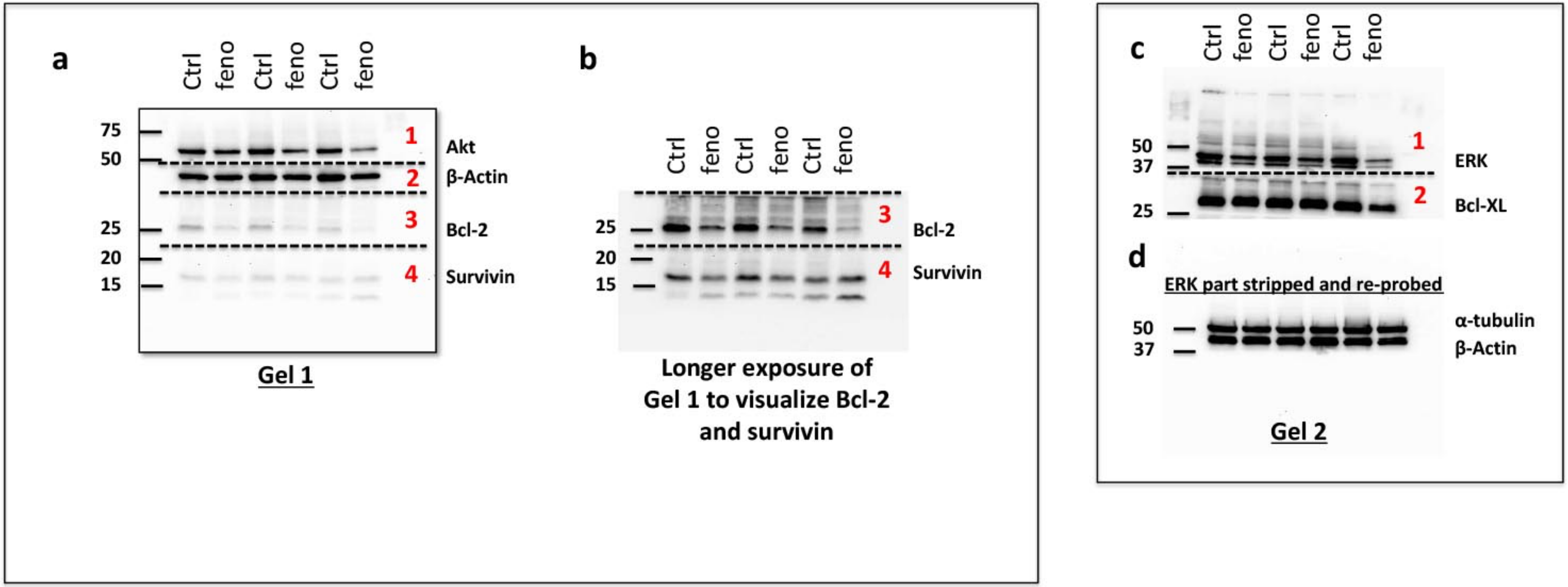

In Run 2 (Gels 1 and 2), Control and fenofibrate-treated cell lysates from 3 independent experiments were prepared and analyzed by western blotting. Following transfer, membrane for Gel 1 was divided into 4 parts and probed for Akt, β-actin, Bcl-2 and survivin (as indicated). Akt and β-actin were readily visualized (a) and a longer exposure was needed to visualize Bcl-2 and survivin (b). Membrane for Gel 2 was divided into 2 parts and probed for ERK and Bcl-XL, which were readily detected (c). The 'ERK part' of the membrane was then stripped and re-probed for α-tubulin and β-actin (d) (antibodies were raised in different species so simultaneous probing was possible).
